# Use of the oral microbiota as screening test to identify children at risk for caries development. A systematic review of longitudinal studies

**DOI:** 10.1111/eos.70069

**Published:** 2026-02-03

**Authors:** Heitor Sales de Barros Santos, Maria Eduarda Lisboa Pagnussatti, Marisa Maltz, Rodrigo Alex Arthur

**Affiliations:** ^1^ Department of Preventive and Community Dentistry Dental School, Federal University of Rio Grande do Sul Porto Alegre Brazil

**Keywords:** biomarkers, dental caries, oral health

## Abstract

This study investigated trends in oral microbiota composition (index test) that could indicate potential candidates to identify children at risk for dental caries development compared with visual/tactile examination (reference test). MEDLINE/PubMed, Web of Science, Embase, Scopus, Lilacs, SciELO, and Google Scholar databases were searched up to September 2025. Methodological quality was assessed by Newcastle‐Ottawa Scale and QUADAS‐2. Qualitative synthesis was performed using all included studies. Thirteen studies that assessed the oral microbiota composition through high‐throughput sequencing platforms were included comprising 740 caries‐free participants at the baseline. *Alloprevotella* spp. and *Megasphaera* spp. were exclusively highly abundant in children who developed caries, whereas *Peptostreptococcus* spp. was exclusively highly abundant in caries‐free children. The diagnostic value of the oral microbiota composition showed specificity, sensitivity, accuracy, and area under the receiver operating characteristics curve ranging from 0.6 to 1.0, 0.75 to 0.90, 0.73 to 0.93, and from 0.51 to 0.94, respectively. High risk of bias was found for the index test. The available evidence does not support the use of oral microbiota composition as a screening test to identify children at risk for caries development. Studies conducted at the species level are likely to provide results with greater sensitivity and specificity, improving risk assessment and understanding of caries‐associated microbiota (PROSPERO CRD42023495648).

## INTRODUCTION

Untreated dental caries remains as a global public health challenge with approximately 63% of children being affected by this condition by the age of 5 years [1, 2]. An increment of caries in deciduous teeth of approximately 11.7% from 1990 to 2019 has been reported, corresponding to 1.15 billion new cases of caries during that period of time [2]. This condition puts public and private care services under pressure for both human and financial resources needed for the management and treatment of this disease. Tooth extraction, extensive restorative and orthodontic treatments, acute and chronic pain as well as hospitalization are some of the consequences of the untreated caries during childhood [1].

Carious lesions result from the imbalance on de‐ and remineralization processes over the life span. Early noncavitated carious lesions are clinically detected only when the mineral loss and the porosity of the subsurface area reach certain level that modifies the optical properties of the enamel surface [3]. If the process is not properly controlled at that time, mineral loss will continue and cavitation might result [4]. Carious lesions are clinically detected by visual‐tactile examination [5], and the lesion activity assessment is an essential part of the diagnosis and of the disease management [6]. Based on the clinical features of the affected hard tissues, the lesions are diagnosed as active, representing an ongoing mineral loss, or as inactive, signaling that the de‐ and remineralization processes at that specific surface have been controlled. Thus, the clinical features of carious lesions may be then used to classify the caries activity profile at individual level [7, 8].

The imbalance on de‐ and remineralization processes is mostly induced by a sugar‐rich diet [9, 10, 11]. Through the metabolism of dietary sugars by saccharolytic oral microorganisms, acid‐ends products keep an acidic environment below the critical pH favoring the mineral loss. At the same time, maintaining an acidic oral environment over time leads to microbial dysbiosis by selection of highly acid‐producers and acid‐tolerant microorganisms that perpetuate the cariogenic‐likely environment [12]. Even though mutans streptococci and lactobacilli have been traditionally associated with the dental caries process [13, 14, 15, 16], the advances on the molecular methods for characterization of microbial communities have shown that the microbiota associated with dental caries is taxonomically more complex. While the oral microbiota of caries‐affected individuals tends to be less diverse compared with those of caries‐free individuals, a greater abundance of *Atopobium* spp., *Bifidobacterium* spp., *Fusobacterium* spp., *Olsenella* spp., *Prevotella* spp., among others, has been reported in the oral cavity of caries‐affected individuals [17]. Enriched metabolic pathways, such as carbohydrate metabolism, phosphotransferase sugar uptake system, ABC transporter systems, among others, seem also to be attributed to the dysbiotic microbial community associated with dental caries [17]. However, the bulk of the evidence about the association between the oral microbiota composition and dental caries comes from cross‐sectional studies and from two distinct groups of individuals: caries‐free and caries‐affected ones [17]. Although this evidence has contributed to expand our knowledge about the microbiology of dental caries to an unprecedented level, the available data do not enable us to clearly identify potential microbial markers of greater risk for caries development.

Dental caries in deciduous dentition predicts the development of this disease during the adolescence [18]. Moreover, dental caries in childhood has been associated with adverse impacts on later oral health‐related quality of life [19]. Thus, early identification of children at higher risk for caries development is desirable allowing both patient‐centered disease management and better resources allocation for control and treatment, particularly for those with the greatest need. Several caries risk assessment tools have been used to estimate the individual risk for caries increment over a certain period of time, but their sensitivity, specificity, and accuracy are limited [20, 21, 22, 23]. Moreover, the performance of caries risk assessment tools has been considered modest and the search for alternative predictors is needed [24]. To date, the past experience of dental caries is still the best and the strongest predictor to estimate the individual risk for caries development [25]. Therefore, the incorporation of new sources of predictors, such as omics‐based ones, have been encouraged in an effort to increase the accuracy of the existing caries risk assessment tools [26, 27].

Considering the contribution that the high‐throughput sequencing methodologies have brought to the comprehension of dental caries health‐to‐disease transitioning process, long‐term follow‐up of the oral microbiota of individuals that are caries‐free at the initial assessment but developed carious lesions over a certain period of time may help to identify early signs of dysbiosis that proceeds the clinical detection of carious lesions. Accordingly, new microbial predictors or microbial markers of the health or of the dental caries status might be identified helping in diagnosing individuals at an increased risk for caries development.

Therefore, this systematic review was designed to investigate trends in oral microbiota composition that could indicate potential candidates for screening test to identify children at risk for dental caries development.

## MATERIAL AND METHODS

### Study protocol

This systematic review of observational longitudinal studies was registered on PROSPERO under CRD42023495648. According to the *Cochrane Handbook for Diagnostic Test Accuracy Systematic Reviews*, screening tests can be reported similarly to diagnostic tests [28]. Thus, this review was reported following PRISMA‐DTA guidelines (Preferred Reporting Items for Systematic Reviews and Meta‐analyses of Diagnostic Test Accuracy Test) [29]. The screening test is defined as one capable of identifying diseases in initial stages in asymptomatic and apparently healthy individuals [28]. For the purpose of this review, asymptomatic and healthy individuals were considered caries‐free according to the initial clinical examination performed by the primary studies.

### Review question and PIT strategy

The research questions to be answered *“*Can the oral microbiota composition be used as screening test to identify children at risk for caries development?” was formulated using the PIT (Population, Index Test, Target Condition) [30] strategy as children (P), oral microbiota composition (I), and dental caries (T). The visual‐tactile clinical examination was considered as reference/standard test for dental caries diagnosis. Children were considered caries‐affected whether they presented noncavitated or frank cavitated lesions.

### Search strategy and study selection

Electronic search was carried out in MEDLINE/PubMed, Web of Science, Embase, Scopus, Lilacs, SciELO, and Google Scholar databases. The search strategies were: MEDLINE/PubMed: ((oral microbiome [All fields]) AND ((prediction [All fields]) OR (predictive [All fields]))) AND ((dental caries [MeSH Terms]) OR (dental caries)); Web of Science: TS = ((oral microbiome) AND ((prediction) OR (predictive)) AND (dental caries)); Embase: “oral microbiome”/exp AND “prediction”/exp OR “predictive value”/exp AND “dental caries”/exp; Scopus: TITLE‐ABS‐KEY ((oral microbiome) AND ((prediction) OR (predictive)) AND (dental caries)); Lilacs: TITLE, ABSTRACT, SUBJECT (dental plaque or saliva AND microbiota AND dental caries); SciELO: ALL INDEXES (dental plaque or saliva) AND (microbiota or microbiome) AND (dental caries) and Google Scholar—allintitle: oral microbiome and dental caries. Search was carried out in December 2023 and updated in September 2025.

### Article selection process and eligibility criteria

Observational longitudinal clinical studies that evaluated whether the oral microbiota composition is able to predict the development of dental caries in children, and without any restriction on language and on the date of publication, and that used high‐throughput sequencing platforms for the identification of microbial communities (targeted to 16S‐rRNA or ITS genes or by using whole metagenome shotgun) were included in this review. The identified records were imported into reference management software (jabref 5.0, JabRef) and duplicate titles were excluded. Records were independently screened by two calibrated researchers (H.S.B.S. and M.E.L.P.) by title and abstract and agreement between then on inclusion/exclusion was high (kappa = 0.9 for both title and abstract screening). Any disagreement between the two researchers was solved by a third researcher (R.A.A.). Letters to editor, systematic and narrative literature reviews, studies carried out using ProbeSeq or gene cloning, and studies with missing data were excluded. All eligible articles were then assessed for full‐text reading. A manual search was also performed on the list of references of the included articles and in all issues of the following journals published between December 2018 and September 2025: *Caries Research*, *Journal of Dental Research*, *Archives of Oral Biology*, *Clinical Oral Investigations*, *Frontiers in Microbiology*, and *Frontiers in Cellular and Infection Microbiology*. All articles that met the eligibility criteria were included.

### Data extraction

Data such as authors, year of publication, country, follow‐up time, number and age of participants, criteria and threshold for dental caries detection, participant´s caries experience, type of clinical sample, sequencing method and sequencing platform, cut‐off point for reporting the microbial relative abundance, potential microbial markers and microbial taxa predictors, specificity, sensitivity, accuracy, and area under the receiver operating characteristics curve (AUROC) were extracted from the included studies and compiled in an Excel spreadsheet. The corresponding author was contacted by email whenever incomplete or inconsistent data were reported. Studies were further excluded if no additional data were provided. All data were independently extracted by two researchers (H.S.B.S. and M.E.L.P.). Any disagreement between the two researchers was solved by a third researcher (R.A.A.).

### Methodological quality assessment of the primary studies

The Newcastle‐Ottawa Scale (NOS) was used to assess the methodological quality of the included studies. Oral microbial composition was considered as “exposure,” whereas dental caries development was considered as “outcome.” The quality of studies was assessed through the following domains which were classified by stars if the criteria were adequately described: (i) Selection (up to 4 stars), (ii) Comparability (1 star), and (iii) Outcome (up to 3 stars). Studies presenting at least 75% of stars (≥6 stars) were graded as “good,” whereas those presenting from 25% to 75% (≥2 and <6 stars) and those presenting less than 25% of stars (<2 stars) were graded as “fair” and “poor,” respectively [31]. Besides, QUADAS‐2 guideline (Quality Assessment of Diagnostic Accuracy Studies Included in Systematic Reviews) [32] was used for quality assessment of those studies which reported sensitivity, specificity, accuracy, and/or AUROC values for the oral microbial composition as a screening test for dental caries development. QUADAS‐2 is structured in four domains as (i) Patient selection, (ii) Index test, (iii) Standard/reference test, and (iv) Patient flow and timing of index and reference tests. New clinical caries lesions through visual‐tactile detection was considered as the standard/reference test. Quality assessment was individually carried‐out by two researchers (H.S.B.S. and M.E.L.P.). Any disagreement in the quality assessment was resolved by a third researcher (R.A.A.).

### Synthesis of evidence

All included studies were descriptively reported for each of the assessed outcomes.

## RESULTS

### Selection of studies

**FIGURE 1:**
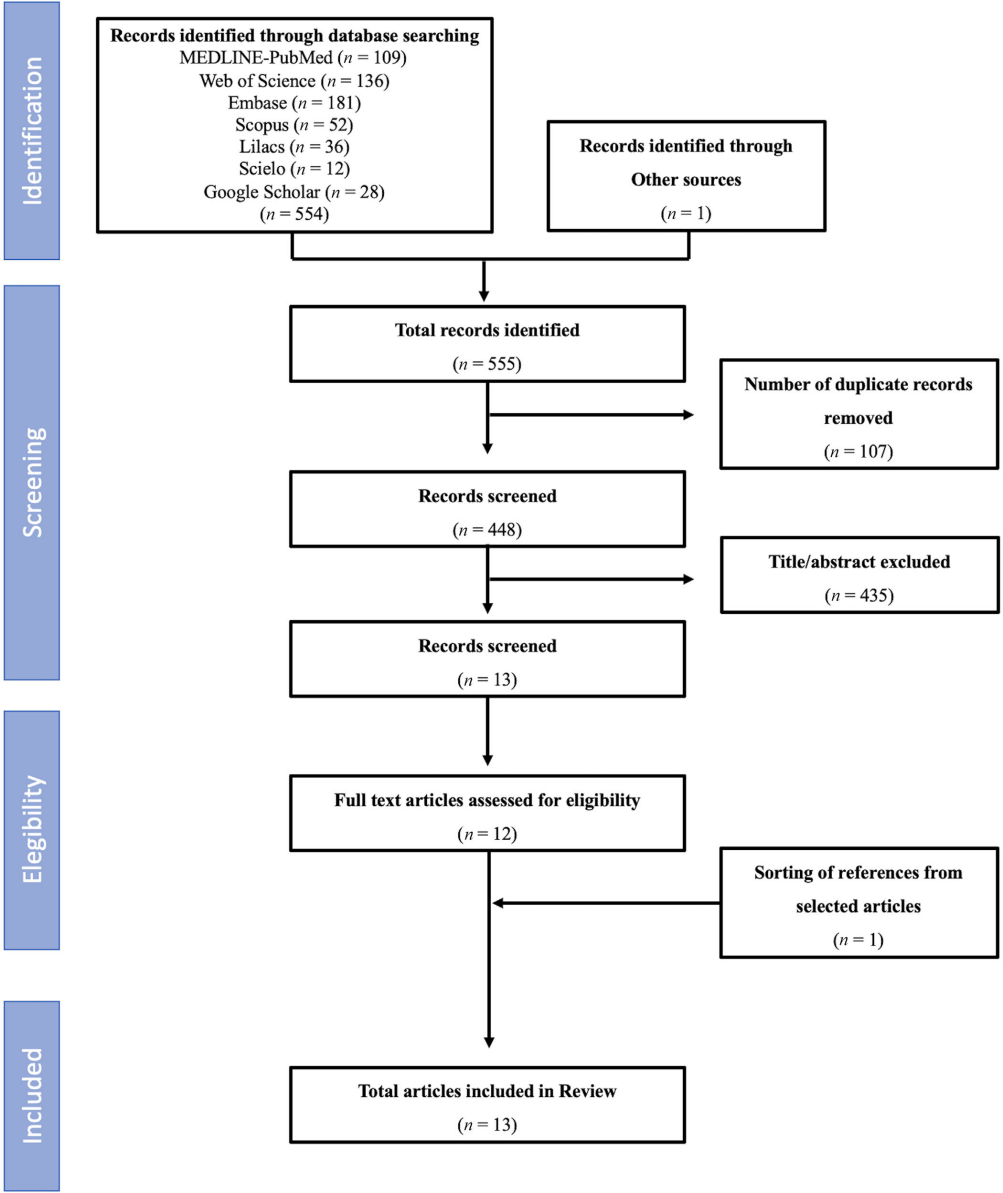
Flow chart of screened eligible and included studies according to PRISMA‐DTA (Preferred Reporting Items for Systematic Reviews and Meta‐analyses of Diagnostic Test Accuracy Test) guideline.

The search strategy retrieved 554 studies being one of them [33] identified through journals screening. After removing duplicates and after screening titles and abstracts, 12 studies were eligible for full reading. One study [34] was manually selected from the reference list of the included articles. Therefore, data were extracted from 13 studies (Figure 1). All selected articles were accessible for full reading.

### Characteristic of the included studies

#### Countries

Most of the included studies were conducted in Asia (*n* = 8; [35, 36, 37, 38, 39, 40, 41, 42]), followed by Europe (*n* = 2; [34, 43]), North America (*n* = 2; [33, 44]), and Australia/Oceania (*n* = 1; [45]).

#### Number and age of participants

A total of 817 participants were evaluated, aged from 2 months to 9 years at the baseline. In eight studies [33, 34, 36, 38, 39, 40, 44, 45], all children were caries‐free at baseline, whereas in five studies [35, 37, 41, 42, 43], caries‐active children were also assessed at baseline in addition to the cohort of caries‐free children. From the total of 740 caries‐free children at baseline, 44.2% remaining caries‐free at all follow‐up times. In one study [35], 13 children already had previous caries experience at the baseline and 7 of these showed recurrent lesions, whereas the remaining showed nonrecurrent lesions over 12 months. In another study [37], caries development over 24 months was observed in all 12 children that already had caries experience at baseline (Table S1).

#### Type of clinical sample and protocol of sample collection

In all but two studies [38, 42], the oral microbiota composition was assessed on saliva samples, being unstimulated saliva collected in five studies [36, 40, 41, 43, 45] or stimulated saliva collected in three studies [35, 39, 44], respectively. In two studies, the oral microbiota composition was exclusively assessed on supragingival plaque [38, 42]. In another study, both unstimulated and stimulated saliva were collected [34], and in another two studies [33, 37], supragingival plaque and unstimulated saliva were used to assess the microbiota composition. Drooling of unstimulated saliva was the method most commonly used for sample collection [36, 37, 40, 41, 45] followed by oral swabbing [33, 43] and pumping [34]. Collection of stimulated saliva was performed by chewing [34, 39], expectoration [35], or pumping [44]. Saliva samples were collected after 1 h of fasting and after 12 h since the last tooth cleaning, or after 2 h of fasting [34, 37, 39]. In two studies, collection was performed under fasting and without previous toothbrushing [36, 41]. No other clear information regarding saliva collection was provided by the other studies. Plaque samples were collected from all available tooth surfaces [37], from all sound surfaces [38], from all buccal surfaces [33], or from a pool of molars and incisors surfaces in caries‐free teeth and from enamel surfaces of carious teeth or from adjacent enamel in caries active children [42], following 1–2 h of fasting [37, 38, 42] or after 12 h since the last tooth cleaning [37, 38] (Table S1).

#### Threshold for carious lesion detection

In five studies, the threshold for detecting dental caries was the presence of noncavitated lesions [33, 34, 38, 40, 44], whereas the presence of frank cavitation was the threshold in other six studies [35, 36, 37, 39, 43, 45]. In the remaining two studies, the threshold for caries detection was not clearly described [41, 42] (Table S1).

#### Sequencing platforms and threshold level for the taxonomic relative abundance

In respect to the sequencing platform, Illumina was the most used one [33, 34, 35, 36, 38, 40, 41, 44], followed by pyrosequencing [37, 39, 43], PacBio [42], and Ion torrent [45]. The 16S‐rRNA V3‐V4 region was sequenced by most of the studies [34, 35, 36, 38, 40], followed by V1–V3 [37, 39, 44] and by V4 [33, 41, 45] regions. A universal bacterial primer was used for microbial community characterization in one study [43]. The 16S‐rRNA sequenced region was not reported in one study [42]. None of the included studies assessed the fungal component of the clinical samples. The threshold for reporting the microbial relative abundance was informed by seven studies: in five of them, the threshold was ≥1% [34, 35, 38, 39, 43], whereas in other two studies, the threshold was 0.1% [44] or 0.01% [33]. The remaining studies did not inform about the threshold used for reporting the microbial relative abundance (Table S1).

#### Potential microbial markers

In total, the abundance of 52 bacterial genera was reported as being statistically different between those children who remained caries‐free and those who developed carious lesions within the follow‐up periods. Those genera belonged to nine phyla (Actinobacteria, Bacteroidetes, Firmicutes, Fusobacteria, Proteobacteria, Candidate Phyla Radiation, Chloroflexi, Spirochaetes, and Synergistetes). Most of the differently abundant genera (*n* = 22; 42.3%) belonged to Firmicutes, followed by Proteobacteria (*n* = 12; 23.1%), Actinobacteria (*n* = 7; 13.5%), and Bacteroidetes (*n* = 5; 9.6%) being the remaining ones (*n* = 6) belonging to the other reported phyla (Figure S1). Several bacterial genera seemed to be more frequently found in children who remained caries‐free, whereas other genera were more frequently found in caries‐affected children. All these genera were reported in each of the condition by at least one study (Table S1 and Figure S1). *Bergeyella* spp., *Haemophilus* spp., *Neisseria* spp., *Peptostreptococcus* spp., and *Porphyromonas* spp. were reported as being more abundant in children who remained caries‐free, while *Alloprevotella* spp., *Corynebacterium* spp., *Megasphaera* spp., *Prevotella* spp., *Rothia* spp., *Streptococcus* spp., *Selenomonas* spp., and *Veillonella* spp. were reported as being more abundant in caries‐affected individuals by at least three studies. Among these genera, *Peptostreptococcus* spp. was only found in individuals who remained caries‐free, whereas *Alloprevotella* spp. and *Megasphaera* spp. were found only in caries‐affected individuals. Other genera, such as *Actinomyces* spp., *Campylobacter* spp., *Capnocytophaga* spp., *Fusobacterium* spp., *Kingella* spp., and *Leptotrichia* spp., seemed to be frequently found in both caries‐free and in caries‐affected individuals (Figure S1). A similar oral microbiota composition among caries‐free and caries‐affected individuals was reported by only one study [34].

#### Bacterial taxa predictors

Seven studies used random forest models to identify potential microbial markers associated with the oral health or with the early‐stage of dysbiosis that could predict the caries development (Table 1).

**TABLE 1:** Summary of cohort studies that evaluated the diagnostic accuracy of the oral microbiome.

Study (year)	Bacterial taxa predictors	Sp	Ss	Acc	AUROC
Zhu et al. [35]	**Caries development after 12 months**: Higher abundance of *Capnocytophaga* spp., *Fusobacterium* spp., and *Leptotrichia* spp. and lower abundance of *Prevotella* spp. at baseline	NR	NR	NR	RFM Higher abundance of *Capnocytophaga* spp., *Fusobacterium* spp., and *Leptotrichia* spp. and lower abundance of *Prevotella* spp. at the baseline (0.952 for all 4 genera) For individual taxa: *Capnocytophaga* spp. (0.833) *Fusobacterium* spp. (0.857) *Leptotrichia* spp. (0.786) *Prevotella* spp. (0.833)
Teng et al., [37]	**Caries‐free status**: greater abundance of *Corynebacterium durum, S. infantis, S. mitis, S. oralis, S. pneumoniae* at baseline **Caries development/progression**: greater abundance of *D. invisus, Eikenella corrodens, F. nucleatum, Leptotrichia_IK040, K. oralis, subsp. animalis, M. micronuciformis, P. denticola, P._DO039, P. histicola, P. muculosa, P. pallens, P. salivae, P. veroralis, S. veroralis*, *S. mutans, V. atypica, V. dispar, V. parvula* at baseline	NR	NR	NR	RFM: **Caries‐affected** **Saliva**: *Prevotella DO039* (0.657) *P. histicola* (0.73) *P. pallens* (0.726) *P. salivae* (0.75) *P. veroralis* (0.7) *S. mutans* (0.633). *P. mucolosa* (0.711) *P. veroralis* (0.694) *S. noxia* (0.729) *S. mutans* (0.69) *V. atypica dispar parvula* (0.715). **Caries‐free** **Saliva**: *C. durum* (0.657) *S. mitis pneumonia infantis oralis* (0.687).
H. Xu et al. [38]	? Not possible to find the data	RFM Baseline 1.0	RFM Baseline 0.833	RFM 0.931	RFM 0.947
Raksakmanut et al. [40]	**Caries‐free/remained caries‐free**: greater abundance of *Campylobacter concisus*, *Leptotrichia* sp. HMT 215, *P. nanceiensis* and *P. melaninogenica* at baseline	RFM/ LEfSe 0.80	RFM/ LEfSe 0.80	RFM/ LEfSe 0.80	RFM/ LEfSe 0.80
Yu et al., [41]	**Prediction model 1 for caries development**: *Atopobium* spp., *Butyrivibrio* spp., *Capnocytophaga* spp., *Eubacterium_nodatum_group, Kingella* spp., *Lautropia* spp., *Oribacterium* spp., *Selenomonas* spp.	NR	NR	NR	RFM Prediction Model 1. (0.517)
	**Prediction model 2 for caries development**: *Atopobium* spp.*, Butyrivibrio* spp.*, Megasphaera* spp.*, Mogibacterium* spp.*, Prevotella* spp. *and Stomatobaculum* spp.	NR	NR	NR	RFM Prediction Model 2. (0.896)
Ho et al., [42]	**Caries‐free**: higher abundance of *human oral bacterium C730* at baseline **Caries development**: higher abundance of *Porphyromonas sp. oral taxon 278 str. W7784* and *Selenomonas sp. oral taxon 137* at baseline	*Human oral bacterium C730* = 0.61 *Porphyromonas sp. oral taxon 278 str. W7784* = 0.75 *Selenomonas sp. oral taxon 137* = 0.61	*Human oral bacterium C730* = 0.88 *Porphyromonas sp. oral taxon 278 str. W7784* = 0.9 *Selenomonas sp. oral taxon 137* = 0.750	NR	RFM *Human oral bacterium C730* = 0.710 *Porphyromonas sp. oral taxon 278 str. W7784* = 0.796 *Selenomonas sp. oral taxon 137* = 0.617
Grier et al. [44]	**Caries‐free**: greater abundance of *Neisseria subflava, Pseudomonas* spp., and *V. parvula* at baseline	RFM Baseline 0.600	RFM Baseline 0.806	RFM Baseline 0.732	RFM Baseline: 0.71
	**Caries‐affected**: greater abundance *Corynebacterium* spp., *Eikenella* spp., *Kingella* spp., *N. cinerea*, *R. mucilaginosa, P. tanaerae, Selenomonas* spp. and *S. noxia* at baseline	RFM Baseline 0.600	RFM Baseline 0.806	RFM Baseline 0.732	RFM Baseline: 0.71

*Note*: Sp, Ss, Acc, and AUROC were calculated based on taxa predictors.

Abbreviations: Acc, accuracy; AUROC, area under the ROC; LEfSe, linear discriminant analysis effect size; NR, not reported; RFM, random forest model; Sp, specificity; Ss, sensitivity.

Greater abundance of *C. durum* and of *Streptococcus mitis_pneumonia_infantis_oralis* in the saliva at the baseline was found in children who remained caries‐free within 12 months (AUROC of 0.65 and 0.68, respectively) [37]. Greater abundance of *C. concisus, Leptotrichia* sp. HMT 215, *P. nanceiensis*, and *P. melaninogenica* at baseline was also observed in children who remained caries‐free within 12 months (specificity, sensitivity, accuracy, and AUROC of 0.80) [40]. Furthermore, greater abundance of *N. subflava, Pseudomonas* spp., and *V. parvula* was found in the saliva at baseline in those children who remained caries‐free over 24 months [44]. Moreover, higher abundance of human oral bacterium C730 at baseline was also observed in children who remained caries‐free within 12 months (AUROC of 0.71) [42].

A greater abundance of *Prevotella*_DO039, *P. histicola*, *P. pallens*, *P. salivae* in the saliva, of *P. veroralis* and *Streptococcus mutans* in the dental plaque and in the saliva, and of *Dialister invisus*, *Eikenella corrodens*, *F. nucleatum*, *Leptotrichia*_IK040, *K. oralis*, subsp. animalis, *M. micronuciformis*, *P. denticola*, *P. muculosa*, *S. veroralis*, *V. atypica*, *V. dispar*, *V. parvula* in dental plaque at baseline indicated dental caries onset within the following 6 and 12 months (AUROC between 0.64 and 0.75) [37]. Higher abundance of *Capnocytophaga* spp., *Fusobacterium* spp., and *Leptotrichia* spp. and lower abundance of *Prevotella* spp. in the saliva at baseline indicated caries development 6 and 12 months later (AUROC of 0.95) [35]. Greater abundance of *Corynebacterium* spp., *Eikenella* spp., *Kingella* spp., *N. cinerea*, *R. mucilaginosa*, *P. tanaerae*, *Selenomonas* spp., and *S. noxia* in the saliva at baseline indicated caries development within the following 6 and 24 months (specificity, sensitivity, accuracy, and AUROC of 0.60, 0.80, 0.73, and 0.71, respectively) [44]. Higher abundance of *Porphyromonas* sp. oral taxon 278 srt. W7784 and *Selenomonas* sp. oral taxon 137 in the dental plaque at baseline indicated caries development in the following 12 months (AUROC of 0.796 and 0.617, respectively) [42]. In one study [38], the abundance of 11 bacterial taxa in the dental plaque was associated with dental caries development 6 and at 12 months later (specificity, sensitivity, accuracy, and AUROC of 1.0, 0.83, 0.931, and 0.94, respectively) [38].

One study reported that two different combinations of bacteria in the saliva were also able to predict caries development with an AUROC of 0.52 (*Atopobium* spp., *Butyrivibrio* spp*., Capnocytophaga* spp*., Eubacterium_nodatum_group, Kingella* spp*., Lautropia* spp*., Oribacterium* spp., and *Selenomonas* spp.) and an AUROC of 0.90 (*Atopobium* spp*., Butyrivibrio* spp*., Megasphaera* spp*., Mogibacterium* spp*., Prevotella* spp., and *Stomatobaculum* spp.) [41].

In three studies, the abundance of some bacteria was also considered as predictors for caries development. Greater abundance of *Leptotrichia* HOT 417 and *P. histicola* in the saliva and of *Leptotrichia* HOT 417 and *L. wadei* in the dental plaque was observed about 2.5 years before the onset of the carious lesions [33]. Children who developed caries at 9 years of age presented greater abundance of *S. cristatus* in the saliva when they were 24 months and 3 years old [34]. Children who developed caries at 39 or 48.6 months of age presented greater abundance of *Streptococcus mutans* in the saliva from 10 to 20 months before the onset of the carious lesions [45]. However, no specificity, sensitivity, accuracy, and AUROC estimates were provided for these microbial predictors.

#### Assessment of the methodological quality of the primary studies

All but one study [33] was classified as “good” [34, 35, 36, 37, 38, 39, 40, 41, 42, 43, 44, 45] (Table S2). In relation to the Selection domain, none of the studies used a representative exposed cohort since the participants came from a convenience sample, such as hospital users, whereas the exposure was already present at the baseline for five studies [35, 37, 41, 42, 43]. For the Comparability domain, confounding factors (e.g., age, socioeconomic factors, antibiotic use, diet) were not adjusted in one study [33]. For the Outcome domain, one study did not report the reasons for losses in follow‐up [33].

Seven studies were also assessed by QUADAS tool [35, 37, 38, 40, 41, 42, 44] (Table 2). A high risk of bias in patient selection (Domain 1) was found in four studies [35, 37, 41, 42] because two distinct group of individuals (caries‐free and caries‐affected ones) were enrolled at the baseline. Therefore, a high risk of applicability concern was attributed to them. All studies presented high risk of bias related to the index test (Domain 2; oral microbiota composition) because it was not possible to infer what was considered as high/low for taxa abundance. As one study [38] did not clearly show which bacterial predictors were used to estimate sensitivity, specificity, accuracy, and AUROC, a high risk of applicability concern was attributed to it. In relation to the Domains 3 and 4 (reference standard and flow and timing), all studies presented a low risk of bias and of applicability concerns.

**TABLE 2:** Quality assessment of diagnostic accuracy studies by QUADAS tool [32].

Domain	Item	Zhu et al. [35]	Teng et al. [37]	Xu et al. [38]	Raksakmanut et al. [40]	Yu et al. [41]	Ho et al. [42]	Grier et al. [44]
#1: Patient selection	Was a consecutive or random sample of patients enrolled?	No	No	Yes	Yes	No	Yes	Yes
	Was a case–control design avoided?	No	No	Yes	Yes	No	No	Yes
	Did the study avoid inappropriate exclusions?	Yes	Yes	Yes	Yes	Yes	Yes	Yes
	**Could the selection of patients have introduced bias?**	**Yes**	**Yes**	**No**	**No**	**Yes**	**Yes**	**No**
	**Concerns regarding applicability**: Is there concern that the included patients do not match the review question?	**Yes**	**Yes**	**No**	**No**	**Yes**	**Yes**	**No**
#2:Index test	Were the index test results interpreted without knowledge of the results of the reference standard?	Yes	Yes	Yes	Yes	Yes	Yes	Yes
	If a threshold was used, was it prespecified?	No	No	No	No	No	No	No
	**Could the conduct or interpretation of the index test have introduced bias?**	**Yes**	**Yes**	**Yes**	**Yes**	**Yes**	**Yes**	**Yes**
	**Concerns regarding applicability**: Is there concern that the index test, its conduct, or interpretation differ from the review question?	**No**	**No**	**Yes**	**No**	**No**	**No**	**No**
#3: Reference standard	Is the reference standard likely to correctly classify the target condition?	Yes	Yes	Yes	Yes	Yes	Yes	Yes
	Were the reference standard results interpreted without knowledge of the results of the index test?	Yes	Yes	Yes	Yes	Yes	Yes	Yes
	**Could the reference standard, its conduct, or its interpretation have introduced bias?**	**No**	**No**	**No**	**No**	**No**	**No**	**No**
	**Concerns regarding applicability**: Is there concern that the target condition as defined by the reference standard does not match the review question?	**No**	**No**	**No**	**No**	**No**	**No**	**No**
#4: Flow and timing	Was there an appropriate interval between index test(s) and reference standard?	Yes	Yes	Yes	Yes	Yes	Yes	Yes
	Did patients receive the same reference standard?	Yes	Yes	Yes	Yes	Yes	Yes	Yes
	Were all patients included in the analysis?	Yes	Yes	Yes	Yes	Yes	Yes	Yes
	**Could the patient flow have introduced bias?**	**No**	**No**	**No**	**No**	**No**	**No**	**No**

	Risk of bias				Applicability concerns		
Study	Patient selection	Index test	Reference standard	Flow and timing	Patient selection	Index test	Reference standard
Zhu et al. [35]	☹	☹	☺	☺	☹	☺	☺
Teng et al. [37]	☹	☹	☺	☺	☹	☺	☺
Xu et al. [38]	☺	☹	☺	☺	☺	☹	☺
Raksakmanut et al. [40]	☺	☹	☺	☺	☺	☺	☺
Yu et al. [41]	☹	☹	☺	☺	☹	☺	☺
Ho et al. [42]	☹	☹	☺	☺	☹	☺	☺
Grier et al. [44]	☺	☹	☺	☺	☺	☺	☺

*Note*: ☺Low risk; ☹High risk.

## DISCUSSION

The compiled data provided by this systematic review show that there was a trend in the differential abundance of certain bacterial taxa among individuals who remained caries‐free (representing a microbial homeostasis) and those who developed dental caries within a certain period of time. Early signs of microbial dysbiosis, represented by the increased abundance of some taxa, including *Alloprevotella* spp., *Corynebacterium* spp., *Megasphaera* spp., *Prevotella* spp., *Rothia* spp., *Selenomonas* spp., *Streptococcus* spp., and *Veillonella* spp., might be observed in the saliva and in the dental plaque several months [35, 36, 37, 38, 40, 41, 42, 43, 44, 45], and even from 2.5 to 7 years [33, 34, 39] before the onset of the carious lesions. *Alloprevotella* spp. and *Megasphaera* spp. were frequently found and exclusively highly abundant in the oral cavity of individuals who developed dental caries over certain period of time [33, 35, 36], whereas *Peptostreptococcus* spp. was not found in abundance in those individuals [37, 44, 45]. On the other hand, greater abundance of *Bergeyella* spp., *Neisseria* spp., *Peptostreptococcus* spp., and *Porphyromonas* spp. are indicative of homeostasis associated with caries‐free conditions [33, 35, 36, 37, 38, 44, 45]. Since an extensive description of the microbial physiological and functional traits is not within the scope of this review, some specific bacterial traits which might be associated with caries‐free and with dental caries conditions are presented below.

*Bergeyella* spp. and *Porphyromonas* spp. are nonacidogenic and nonsaccharolytic microorganisms [46, 47]. *Bergeyella* spp. are able to convert tryptophan into indole [47]. This conversion, that is based on deamination of tryptophan leading to the production of amine compounds, along with the reduction of nitrate or nitrite by *Haemophilus* spp. and *Neisseria* spp. [48, 49], produce byproducts that act as hydrogen acceptors contributing to the buffering of the oral cavity [50]. In addition, *Haemophilus* spp. are able to produce ammonia from urea (via urease activity) which raises the pH of the dental plaque contributing to the maintenance of oral health. [48, 51]. Some species of *Peptostreptococcus* are asaccharolytic, indole positive and have the capacity to reduce nitrate to nitrite, justifying their participation in health [52].

On the other hand, evidence indicates that *A. rava, C. matruchotii, M. micromuciformis*, *R. dentocariosa*, and *S. noxia* contribute to the maintenance of acidic environments by producing acids from carbohydrate degradation, such as acetic, formic, lactic, and succinic acids [53, 54, 55, 56, 57, 58]. *Prevotella* spp. are considered saccharolytic and their species have the ability to metabolize dextrin, fructose, glucose, inulin, lactose, maltose, mannose, melibiose, rabinose, raffinose, sucrose, xylose, and others, with the final products of metabolism being acetic, succinic, formic, propionic, isobutyric, and isovaleric acids [59]. Symbiotic relationships between species demonstrate that in dual biofilms of *P. denticola* and *S. mutans*, there is a greater production of lactate as a final product of carbohydrate metabolization, in addition to more robust biofilms and a more abundant extracellular matrix when compared to single‐species biofilms [60].

Important microbial–microbial interactions seem also to contribute to the development of a cariogenic environment on tooth surfaces. *F. nucleatum* is considered an intermediate colonizer of tooth surfaces interacting with primary and with late colonizers [61]. *S. mutans* and *F. nucleatum* interact with each other through a pair of RadD‐SpaP adhesins, which provides an additional opportunity for *S. mutans* to colonize cariogenic dental plaque [62]. Besides being acid‐tolerant and acid‐producers, synthesis of extracellular polymers is also a virulence feature of *S. mutans*. Those polymers, as part of dental plaque matrix, facilitate the adhesion of microorganisms to dental surfaces, mediate microbial–microbial co‐adhesion, and become dental plaque more porous which contributes to a greater diffusion of nutrients and acids throughout the dental plaque thickness, exposing dental surfaces to an increased risk for mineral loss [63]. In this sense, the production of extracellular polymers by *F. nucleatum* has also been considered to contribute to the maturation of dental plaque [64]. These cariogenic‐likely traits help to understand the association of the above‐mentioned taxa with dental caries condition. Moreover, the interaction among *Veillonella* species and *S. mutans* leads to the formation of a more robust plaque, since *Veillonella* spp. metabolize the lactic acid produced by *S. mutans* into propionic and acetic acid, which are weaker acids, providing a better controlled acidic environment for the growth of *S. mutans* [65]. *S. sputigena* has the ability to build a unique spatial structure in dental plaque that is similar to honeycomb encapsulating *S. mutans*. Under this condition, the virulence of dental plaque is increased leading to extensive carious lesions development [66].

Some genera, such as *Actinomyces* spp., *Campylobacter* spp., *Capnocytophaga* spp., *Fusobacterium* spp., *Kingella* spp., and *Leptotrichia* spp., seemed to be frequently reported as being more abundant both in caries‐free and in caries‐affected children, which reinforces the need to investigate bacterial diversity at species level. *Actinomyces* spp., specifically *A. viscosus* and *A. naeslundii*, metabolize dietary carbohydrates and degrade intracellular polysaccharides producing acids that create an environment prone to tooth demineralization [67, 68]. *A. johnsonii* and *A. graevenitzi* exhibit acid‐tolerance similar to *S. mutans* and to *S. sobrinus*, well‐known bacteria associated with dental caries development [68, 69, 70]. *C. concisus* was reported as a predictor for children who remained caries‐free [34]. This microorganism contributes to modulate the acidic environment associated with caries development since they metabolize lactic acid and reduce nitrate [71, 72]. *Capnocytophaga* spp. present features compatible with both health and dental caries conditions, such as degradation of nitrate into nitrite and the ability to degrade sugars leading to acid production [51, 73].

Some species of *Kingella* spp. have the ability to ferment carbohydrates and generate acids as a final product of metabolism [74]. However, many species of this genus are also considered commensals in the oral cavity [75], which would justify the presence of this genus in both caries‐free and caries‐active patients. Moreover, although *Leptotrichia* spp. have been more associated with dental caries, greater abundance of *Leptotrichia* HMT 215 was found in caries‐free individuals, while other species such as *Leptotrichia* HOT 417 and *L. wadei*, were found at greater abundance in those children who were caries‐free but developed dental caries [33, 40]. *Leptotrichia* spp. are facultative anaerobes/strict anaerobes and all species have the ability to ferment carbohydrates and produce lactic acid as a final product of metabolism [76]. It is important to highlight that although *Prevotella* spp. have been frequently found as highly abundant in individuals who develop dental caries, differences exist at species‐level: *P. melaninogenica* and *P. nansceiensis* have been found highly abundant in caries‐free while *P. denticola*, *P. histicola*, *P*. DO39, *P. maculosa*, *P. pallens*, *P. salivae*, *P. tannarea*, and *P. verroralis* seem to be more abundant in caries‐affected individuals. Altogether, these findings mean that the search for microbial markers of the health state (homeostasis) and of the early‐stage of dysbiosis should not be focused only on individual bacterial genus but it should assess the microbial diversity at the species level. Several microbial taxa, especially at the species level, need to be considered taken the importance of microbial–microbial interactions and of the species‐specific phenotypes to the development of a cariogenic‐prone environment. The study of Kahharova et al. [33] clearly showed that several species of the same bacterial genera are highly abundant in each of the assessed conditions.

In addition to those bacterial taxa, frequently more abundant in caries‐affected individuals, *D. invisus, E. corrodens* and *K. oralis* were also indicated as potential taxa predictors of dental caries development. *D. invisus* is asaccharolytic [77], and this species has the ability to metabolize amino acids, peptides, and other noncarbohydrate nutrients [78]. As a final product, it generates acetate, lactate, and propionate [79], an alternative to the classical pathway of acid production through the metabolism of carbohydrates, which contribute to the development of dental caries. In clinical studies, *K. oralis* appears to be correlated with *S. mutans*, that is, samples with high levels of *S. mutans* also presented significantly higher levels of *K. oralis* [80, 81]. However, the reason for this association is unknown and it deserves further research. Although frequently found in subgingival dental plaque [82] and although being considered a nitrate‐reducer microorganism [83], *Eikenella* spp. are also found in supragingival plaque of periodontally health individuals [84, 85]. These microorganisms are able to co‐aggregate, through lectin‐mediated interactions, with strains of *Actinomyces*, such as *A. viscosus*, and with *Streptococcus* spp., such as *S. mutans*, highly prevalent cariogenic microorganisms, which could explain its presence in caries associated biofilms [86]. However, the role of these microorganisms under caries‐prone conditions deserves further investigation.

Carious lesion development is the result of an imbalance on the microbiota of the dental plaque (state of dysbiosis) which is mainly induced by frequent intake of a sugar‐rich diet. Acidification of dental plaque due to microbial fermentation of dietary sugars results in reversible and interconnected stages of microbial changes, which ultimately increases the abundance of acidogenic and acid‐tolerant microorganisms, during the course of carious lesions development and before the lesion be clinically detected [12]. Given that an advanced stage of tooth mineral loss is needed for a carious lesion be clinically detectable and an individual therefore diagnosed with dental caries disease [3], the possibility of identifying microbial markers of early signs of dysbiosis that proceeds the clinical detection of carious lesions is desirable. The long‐term goal of such strategy would be the development of a chair‐side screening test to be used in clinical settings to detect those microbial markers allowing the identification of individuals under risk for caries development. This would allow the adoption of interventions aiming at restoring the microbial balance and at controlling the tooth mineral loss.

Based on specificity, sensitivity, accuracy, and on AUROC values, authors have claimed that the oral microbiota has a potential for being used as predictor to identify children at risk for caries development. However, several concerns related to the primary studies need to be considered. Type of clinical sample (unstimulated/stimulated saliva and dental plaque) and condition of sample collection (fasting, tooth surfaces and time points since the last toothbrushing) were highly variable among the included studies. The microbial composition of stimulated and unstimulated saliva is different, and the number of bacterial species is greater in stimulated saliva [87]. Saliva also harbors a bacterial community that is less diverse, less rich and less even compared with dental plaque [37, 88, 89, 90]. This way, the data obtained from saliva as a clinical source of microorganisms must be interpreted with caution since it does not represent the entire complexity of the microbiota of dental plaque. Different fasting times also affect the oral microbiota composition, since an increase in the abundance of some bacterial taxa is observed up to 70 min after a meal intake [91]. Moreover, time since the last toothbrushing exerts a significant effect on microbial composition of dental plaque considering that both microbial complexity and diversity increase during dental plaque growth [92, 93]. Furthermore, differences in the microbiota composition may also be expected among different tooth surfaces [94, 95].

Detailed information about the sample collection protocol, and the region of 16S‐rRNA that was sequenced were often either absent or they were incompletely reported. Similarly, the cut‐off point for reporting the taxa relative abundance was missing in some of the included studies weakening the reliability of sensitivity, specificity, accuracy, and AUROC values. All these above‐mentioned points constitute important sources of variability among the included studies which may have strongly impacted their outcomes and the comparisons that are summarized in this review. While some studies presented AUROC values based on individual microbial taxa [37, 42], groups of microorganisms were considered in the estimates of other studies [35, 40, 41, 43]. Moreover, in one study, it was not possible to find which bacterial taxa were considered for the AUROC estimate [38]. Therefore, considering the differences among the studies and the concerns described above, a meta‐analysis was not performed.

It is therefore important to highlight that data reporting was highly heterogeneous among the included studies. In one study [38] it was not possible to identify which microorganisms were considered predictors for dental caries. In another one, a statistical analysis comparing taxa abundance among caries‐free and caries‐affected individuals was missing [41]. In one study, a statistical analysis comparing taxa abundance among caries‐free and caries‐affected individuals was missing [41]. As different follow‐up periods and different time points for sample collection were adopted in the primary studies, it was not possible to estimate precisely how early the dysbiosis can be detected in the oral cavity before the onset of the carious lesions. Contingency tables were not presented and further analysis of the data to better estimate the overall diagnostic value of the oral microbiota as a screening test was not possible. We also noted that confounding factors, such as dietary and oral hygiene habits were not reported by any of the included studies. These shortcomings highlight the need for highly standardized protocols not only for experimental design and data collection but also for data reporting. It is urged that microbiome data be reported as clearly as possible and that the raw data be available in public repositories allowing researchers to explore them in complementary analysis [17].

Only seven studies evaluated the diagnostic value of the oral microbiota composition in the identification of children under risk for caries development [35, 37, 38, 40, 41, 42, 44]. Regarding the index test, all of them were graded as high risk of bias because there was not a clear cut‐off point to estimate the relative abundance of the bacterial predictors. This limitation has a detrimental effect on clinical applicability considering that knowing the level of microbial abundance that is compatible with health condition or, conversely, that is indicative of dysbiosis, is desirable. Applicability concerns regarding patient selection were also observed in four studies [35, 37, 41, 42].

While the use of oral microbiota composition as a screening test to predict caries development has shown reasonable sensitivity, specificity, accuracy, and AUROC values, the bulk of the evidence comes from a limited number of studies. Moreover, the included studies presented methodological heterogeneity and high risk of bias. Although a trend has been found in the differential abundance of certain bacterial taxa among individuals who remained caries‐free and those who developed dental caries, the diagnostic value of oral microbiota composition as a screening test to predict caries is still unknown. Moreover, bacteria within the same genus can exhibit different abilities, leading some species to be associated with dental caries while others are not. For this reason, genus‐level data are not enough for reliable prediction. Studies conducted at the species level are likely to provide results with greater sensitivity and specificity, improving risk assessment and understanding of caries‐associated microbiota. Altogether, the bulk of the evidence and the limitations pointed out above lead to the conclusion that it is not possible to use of oral microbiota composition as a screening test to identify children at risk for caries development.

As an inherent limitation of the systematic reviews, we acknowledge that the data presented here might be limited by the literature retrieved by the chosen search strategy. However, to retrieve relevant and potential eligible studies, we have performed the literature search in six important databases (MEDLINE/PubMed, Web of Science, Embase, Scopus, Lilacs, and SciELO) and also in the gray literature (Google Scholar). Besides, a hand‐search in the reference list of the included studies as well as in all issues of six journals was performed in an effort to maximize the inclusion of relevant studies that eventually were not retrieved by the search strategy, but only one study was retrieved by this additional search. This suggests that the search strategy used in this review was able to retrieve the majority of the studies. In view of the characteristics and limitations of the retrieved studies, we hypothesize that the inclusion of any other study would neither change the conclusion nor the concerns arisen by this review.

Future studies need to characterize the oral microbiota composition at species‐level in order to indicate stronger markers associated with caries‐free or with dental caries conditions. Additionally, other microorganisms than bacteria, especially the fungal component, should be taken into account, as there are important inter‐kingdom interactions observed in both healthy and caries conditions. [96]. The development of robust artificial intelligence based‐algorithms may help in the identification of several taxa/species predictors. While other conclusions are precluded, it is expected that further longitudinal studies unravel which group of bacterial species could be clinically considered to increase the accuracy of the existing caries risk assessment tools helping in the identification of children at risk for caries development.

## AUTHOR CONTRIBUTIONS

**Conceptualization**: Arthur RA, Santos HSB. **Formal analysis**: Santos HSB, Pagnussatti MEL, Arthur RA. **Investigation**: Santos HSB, Pagnussatti MEL, Arthur RA. **Methodology**: Santos HSB, Pagnussatti MEL, Arthur RA. **Writing—original draft**: Santos HSB, Maltz M, Arthur RA. **Writing—review and editing**: Santos HSB, Maltz M, Arthur RA.

## CONFLICT OF INTEREST STATEMENT

The authors declare no conflicts of interest.

## Supporting information



Supporting information
