# Supplementary material for: Use of the oral microbiota as screening test to identify children at risk for caries development. A systematic review of longitudinal studies
Source: Eur J Oral Sci. 2026 Feb 3;134(2):e70069. doi: 10.1111/eos.70069 (PMC12976821; doi:10.1111/eos.70069)
Supplement: Supplementary file 1 — Supporting information [file EOS-134-e70069-s001.pdf]

SUPPORTING INFORMATION

Use of the oral microbiota as screening test to identify children at risk for caries development. A systematic review of longitudinal studies

SANTOS HSB, PAGNUSSATTI MEL, MALTZ M, ARTHUR RA.

Dental School, Federal University of Rio Grande do Sul (UFRGS), Porto Alegre, Brazil

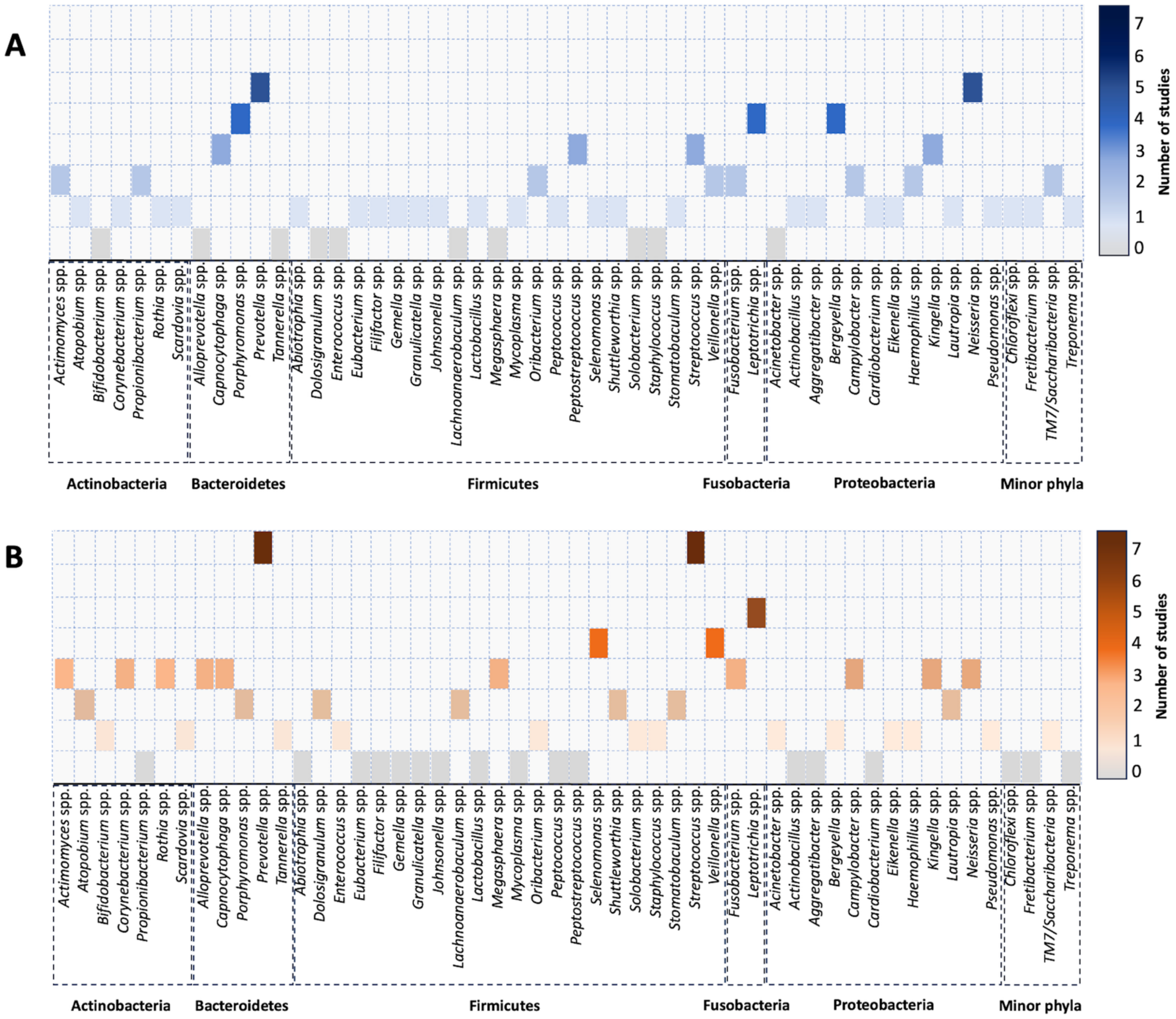

Figure S1: Number of studies reporting greater bacterial abundance in caries-free (A) and in caries-affected (B) individuals. Only taxa whose abundance was statistically higher at each condition was included in this figure. Different shades from light to dark colors indicate an increased number of studies. Grey color means "zero" study.

Table S1. Characteristics of study participants, caries experience and lesion detection threshold, samples used for microbial analysis, sequencing method, cut-off point for reporting relative abundance and potential microbial markers of health and dental caries onset.

Study<br>Contry	Participants (n)<br>[Age at baseline; mean ± sd]	Caries experience<br>(carious lesion detection threshold)	Clinical sample	Method<br>[Cut off point]*	Potential Microbial markers
**Kahharova et al.** [33]<br><br>USA<br><br>(follow-up periods: 1, 2.5, 4 and 6.5 years)	Caries-free/remained caries-free (50)<br><br>Caries-free transitioning to caries-affected at 2.5 years (n=75)*<br><br>Caries-free transitioning to caries-affected at 4 years (n=70)*<br><br>Caries-free transitioning to caries-affected at 6.5 years old (n=38)*<br><br>*remained caries-free until de preceding time points<br><br>[from 1 year-old at baseline to 6.5 years old at the last follow-up)	Caries-free/remained caries-free: ICDAS=0<br><br>At 1 year follow-up:<br>ICDAS≥ 1 (n=2)<br><br>At 2.5 years follow-up:<br>ICDAS≥ 1 (n=76)<br>ICDAS≥ 3 (n=13)<br><br>At 4 years follow-up:<br>ICDAS≥ 1 (n=127)<br>ICDAS≥ 3 (n=52)<br><br>At 6.5 years follow-up:<br>ICDAS≥ 1 (n=107)<br>ICDAS≥ 3 (n=71)<br><br>(non-cavitated lesions)	Unstimulated Saliva (swabbing)<br><br>Supragingival plaque (pooled from all buccal surfaces)<br><br>Both saliva and dental plaque were collected at 1, 2.5 and at 4 years of follow-up	16S-rRNA (V4) Illumina<br><br>[0.01%]	<u>**Saliva:**</u><br>*Comparison between 4 years-old caries-free and caries-affect children when they were 1 year-old (at baseline):<br>**-Caries-free/remained caries-free:** greater abundance of *Bergeyella* spp. (2 stains), *Gemella* spp. (2 strain), *Haemophilus* spp. (1 strain), *Porphyromonas* spp. (3 strains) and of *Streptococcus* spp. (7 strains) at baseline<br>**-Caries-affected:** greater abundance of *Actinomyces* spp. (3 strain), *Alloprevotella* spp. (1 strain), *Enterococcus* spp. (1 strain), *Megasphaera* spp. (1 strain), *Rothia* spp. (1 strain), *Streptococcus* spp. (6 strain) and of *Veillonella* spp. (14 strains) at baseline<br><br>*Comparison between 6.5 years-old caries-free and caries-affect children when they were 4 year-old:<br>**-Caries-free/remained caries-free:** no differentially abundant taxa when they were 4-years old

					**-Caries-affected:** greater abundance of *Leptotrichia* spp. (10 strains) and of *Prevotella* spp. (9 strains) when they were 4-years-old
**Kahharova et al.** [33] cont.	Caries-free/remained caries-free (50)<br><br>Caries-free transitioning to caries-affected at 2.5 years (n=75)*<br><br>Caries-free transitioning to caries-affected at 4 years (n=70)*<br><br>Caries-free transitioning to caries-affected at 6.5 years old (n=38)*<br><br>*remained caries-free until de preceding time points<br><br>[from 1 year-old at baseline to 6.5 years old at the last follow-up)	Caries-free/remained caries-free: ICDAS=0<br><br>At 1 year follow-up:<br>ICDAS≥ 1 (n=2)<br><br>At 2.5 years follow-up:<br>ICDAS≥ 1 (n=76)<br>ICDAS≥ 3 (n=13)<br><br>At 4 years follow-up:<br>ICDAS≥ 1 (n=127)<br>ICDAS≥ 3 (n=52)<br><br>At 6.5 years follow-up:<br>ICDAS≥ 1 (n=107)<br>ICDAS≥ 3 (n=71)<br><br>(non-cavitated lesions)	Unstimulated Saliva (swabbing)<br><br>Supragingival plaque (pooled from all buccal surfaces)<br><br>Both saliva and dental plaque were collected at 1, 2.5 and at 4 years of follow-up	16S-rRNA (V4) Illumina<br><br>[0.01%]	**<u>Dental plaque</u>:**<br>*Comparison between 4 years-old caries-free and caries-affect children when they were 2.5 years-old:<br>**-Caries-free/remained caries-free:** greater abundance of *Abiotrophia* spp. (3 strains), *Actinomyces* spp. (1 strain), *Aggregatibacter* spp./*Haemophilus* spp. (1 strain), *Bergeyella* spp. (1 strain), *Haemophilus parainfluenzae* (2 strains), *Kingella denitrificans/ Neisseria elongate* (1 strain), *Lautropia* spp. (5 strains), *Neisseria* spp. (15 strains) and of *Streptococcus* spp. (4 strains) when they were 2.5 years-old<br>**-Caries-affected:** greater abundance of *Actinomyces* spp. (5 strains), *Capnocytophaga* spp. (1 strain), *Corynebacterium* spp. (10 strains), *Kingella* spp. (2 strains), *Lachnoanaerobaculum* spp. (3 strains), *Leptotrichia* spp. (31 strains); *Neisseria* spp. (3 strains), *Stomatobaculum* spp. (1 strain), *Streptococcus* spp. (13 strains)

					and of *Veillonella* spp. (22 strains) when they were 2.5 years-old.
**Dzidic et al.** [34]<br><br>Sweden<br><br>(follow-up periods: 3, 6, 12 and, 24 months and 7 years)	Caries-free/remained caries-free at 9 years-old (46)<br><br>Caries-free/transitioning to caries-affected at 9 years-old (33)<br><br>[3, 6, 12 and 24 months old and 7 years old]	NR<br><br>(non-cavitated lesions)	Unstimulated saliva (collected by a hand pump; after 2h fasting) collected at 3, 6, 12 and 24 months<br><br>Stimulated saliva collected at 7 years follow-up (chewing)	16 rRNA (V3 – V4) Illumina<br><br>qPCR for *S. dentisani* quantificati-on<br><br>[1%]	There were no differences on general microbial diversity and on taxa abundance between children staying caries-free and children developing caries at 9 years-old
**Zhu et al.** [35]<br><br>China<br><br>(follow-up periods: 6 and 12 months)	Caries-Free/remained caries-free (15)<br>[mean of 55.5 ± 6.66 months]<br><br>Caries affected*:<br>-ECC development (caries recurrence over 12 months) (7)<br>[mean of 54.7 ± 4.54 months old]<br><br>-non caries recurrence over 12 months (6)	Caries-free/remained caries-free: ds=0 (throughout all time points)<br><br>Caries-affected:<br>-ECC development: ds:1-4 (at 6 months) and ds: 2-6 (at 12 months)<br><br>-non-caries recurrence over 12-months: ds:0 (at 6 months) and ds:0 (at 12 months)<br><br>(frank cavitated lesion)	Stimulated saliva (expectoration) collected after water rinse	16S-rRNA (V3-V4) Illumina Miseq<br><br>[>1%]	**Caries-affected:** *Actinomyces* spp., *Alloprevotella* spp., *Campylobacter* spp., *Capnocytophaga* spp., *Corynebacterium* spp., *Fusobacterium* spp., *Haemophilus* spp., *Kingella* spp., *Lautropia* spp., *Leptotrichia* spp., *Neisseria* spp., *Porphyromonas* spp., *Prevotella* spp., *Rothia* spp., *Streptococcus* spp., *Veillonella* spp. dominated the communities in caries-affected individuals at baseline (for both ECC-development and for non-caries recurrence)<br><br>Higher abundance of *Capnocytophaga* spp., *Fusobacterium* spp., and

	[mean of 58.7 ± 4.24 months old]<br><br>*all children were caries-free at baseline but had past experience of caries				*Leptotrichia* spp. and lower abundance of *Prevotella* spp. at baseline (comparison between ECC versus non-caries recurrence)
**Xu et al.** [36]<br><br>China<br><br>(follow-up periods: 6, 12, 18 and 24 months)	Caries-free/remained caries-free (11)<br><br>Caries-free/transitioning to caries-affected (12)<br><br>[mean of 47.5 ± 2.2 months old at baseline]	Caries-free – (dfs=0)<br><br>Caries-affected – (dfs average 4.83)<br><br>(frank cavitated lesion)	Unstimulated saliva (drooling; collected in the morning wo. toothbrushing and under fasting]	16rRNA (V3-V4) Illumina MiSeq<br><br>[NR]	**Caries-free/remained caries-free:**<br>at 6 months: greater abundance of *Bergeyella* spp., *Capnocytophaga* spp., *Haemophilus* spp.,*Neisseria* spp., *Porphyromonas* spp., and *Streptococcus* spp; at 12 months: greater abundance of *Actinobacillus* spp., *Filifactor* spp., and *Peptococcus* spp; at 18 months: greater abundance of *Eikenella* spp; at 24-months: greater abundance of *Actinobacillus* spp., *Bergeyella* spp., *Fretibacterium* spp., *Haemophilus* spp., *Mycoplasma* spp., and *Propionibacterium* spp.<br><br>**Caries-affected:**<br>at 6 months: greater abundance of *Atopobium* spp., *Megasphaera* spp., *Prevotella* spp., *Selenomonas* spp., and *Veillonella* spp.; at 12 months: greater abundance of *Dolosigranulum* spp; at 18 months: greater abundance of *Atopobium* spp., *Megasphaera* spp., and *Veillonella* spp.; at 24-months: greater abundance of *Alloprevotella* spp., *Atopobium* spp., *Lautropia* spp.,

					Megasphaera spp., *Selenomonas* spp., and *Veillonella* spp.
**Teng et al.,** [37]<br><br>China<br><br>(follow-up periods: 6, 12 and 24 months)	Caries-free/remained caries-free (17)<br><br>Caries-free/transitioning to caries-affected (21)<br><br>Caries-affected/carious lesions development (12)<br><br>[from 42 to 63 months old at baseline]	Caries-free/remained caries-free: dmfs=0 (throughout all time points)<br><br>Caries-free/transitioning to caries-affected:<br>dmfs= 3.2±1.8 (12 months)<br><br>Caries-affected/carious lesions development:<br>dmfs= 16.72 ±12.41 (12 months)<br><br>(frank cavitated lesion)	Supragingival plaque (pool of all available surfaces; collection by toothbrushing; after 1h fasting and 12h w.o toothbrushing)<br><br>Unstimulated saliva (drooling; same condition described for plaque collection)	16S-rRNA (V1-V3) Roche 454<br><br>[NR]	**Caries-free/remained caries-free (plaque):** greater abundance of: *Bergeyella_602D02, Campylobacter_gracilis, Cardiobacterium_valvarum,Fusobacterium_nucleatum_subsp._polymorphum,Kingella_denitrificans,Leptotrichia_BU064,Neisseria_falva_mucosa_pharyngis, Neisseria_flavescens, Prevotella_loescheii,Streptococcus_mitis_pneumoniae_infantis_oralis.*<br><br>**Caries-free/remained caries-free (saliva):** greater abundance of: *Bergeyella 602D02, Eubacterium sulci infirmum, Granulicatella_elegans, Leptotrichia BU064, Neisseria flavescens, Neisseria_meningitidis_polysaccharea, Peptostreptococcus stomatis, Porphyromonas catoniae, Porphyromonas CW034, Prevotella melaninogenica, Prevotella oral taxon 299, Streptococcus gordonii, Streptococcus parasanguinis oralis, TM7 oral taxon 352, Veillonella atypica dispar parvula.*

**Xu et al.** [38]<br><br>China<br><br>(follow-up periods: 6 and 12 months)	Caries-free/remained caries-free at both 6 and at 12 months-follow-up (19)<br>[3 years-old at baseline]<br><br>Caries-free/ transitioning to caries affected at 6 months and at 12-months follow-up (10)<br>[3 years-old at baseline]	Caries-free/remained caries-free: dmfs=0 (throughout all time points)<br><br>Caries-free/transitioning to caries-affected: number of decayed teeth (dt) = 2.0 ± 1.1 at 6-months and 5.4 ± 3.0 at 12-months follow-up<br><br>(non-cavitated lesion)	Supragingival plaque (from sound enamel surfaces; after 2h fasting; 12h wo toothbrushing)	16S-rRNA (V3-V4) Illumina Miseq<br><br>[3%]	**Caries-free/remained caries-free:**<br>**0 m-** Greater abundance of *Capnocythophaga* spp., *Kingella* spp., *Neisseria* spp., *Selenomona*s spp.<br>**6 months-** Greater abundance of *Aggregatibacter* spp., *Neisseria* spp., *Prevotella* spp., *Treponema* spp.<br>**12 months-** Greater abundance of *Capnocytophaga* spp., *Johnsonella* spp., *Neisseria* spp., *Saccharibacteria*_genera,incetae_sedis<br><br>**Caries affected:**<br>**0 m-**Greater abundance of *Campylobacter* spp., *Fusobacterium* spp., *Oribacterium* spp., *Prevotella* spp., Saccharibacteria_genera, incetae_sedis, Solobacterium, *Streptococcus* spp.<br>**6 months-** Greater abundance of *Leptotrichia* spp., *Megasphaera* spp.<br>**12 months-**Greater abundance of *Acinetobacter* spp., *Actinomyces* spp., *Dolosigranulum* spp., *Oribacterium* spp., *Pseudomonas* spp.
**Kim et al.** [39]<br><br>Korea	Caries-free/remained caries-free (12)<br>[8.67 ± 1.87 years-old]	NR<br><br>(frank cavitated lesion)	Stimulated saliva (collected after water rinse; chewing; after 2h fasting)	16rRNA (V1-V3) 454 GS Junior	**Caries-free/ remained caries-free:** greater abundance of *Oribacterium* spp. (at baseline) and of *Actinomyces*

(follow-up period: 4 years)	Caries-free/transitioning to caries-affected (12) [9.33 ± 1.67 years-old]			[>1%]	spp., *Atopobium* spp., and *Leptotrichia* spp. (at follow-up) (p<0.1)<br><br>**Caries-Affected (at follow-up):** greater abundance of *Streptococcus* spp. (both at baseline and at the follow-up)(p<0.1)
**Raksakmanut et al.** [40]<br><br>Thailand<br><br>(follow-up period: 1 year)	Caries-Free/remained caries-free (10) [12.7 ± 1.3 months old]<br><br>Caries-free/transitioning to caries-affected (non-cavitated lesions (10) [12.6 ± 1.1 months old]<br><br>Caries-free/transitioning to caries-affected (non-cavitated and cavitated lesions) (10) [12.4 ± 0.5 months old]	Caries-Free: dmfs=0<br><br>Caries-free/transitioning to caries-affected (non-cavitated lesions: dmfs=6.1±2.1<br><br>Caries-free/transitioning to caries-affected (non-cavitated and cavitated lesions) dmfs=8.6±4.7<br><br>(non-cavitated lesions)	Unstimulated saliva (drooling)	16S-rRNA (V3-V4) Illumina MiSeq<br><br>[NR]	**Caries-free/remained caries-free:** greater abundance of *Campylobacter concisus, Leptotrichia* sp. HMT 215, *Prevotella melaninogenica and Prevotella nanceiensis* at baseline
**Yu et al., [41]**<br><br>China<br><br>(follow-up period: 3	Caries-Free/remained caries-free: (6)<br><br>Caries-free/transitioning to caries affected (18)	Caries-free/remained caries-free = dmft = 0<br><br>Caries-free/transitioning to caries affected = dmft > 0<br><br>Caries affected = dmft ≥ 6	Unstimulated saliva (drooling; collected in the morning wo. toothbrushing and under fasting]	16S- rRNA (V4) Illumina MiSeq<br><br>[NR]	**Caries-free/remained caries-free:** greater abundance of *Haemophilus* spp., *Neisseria* spp., *Rothia* spp., *Streptococcus* spp. at baseline

and 6 months)	Caries affected (20)<br><br>[from 3 to 5 years-old]	[it is not clear whether non-cavitated lesions were included]			**Caries-affected**: greater abundance of *Leptotrichia* spp., *Prevotella* spp., *Veillonella* spp. at baseline<br><br>The abundance of these microorganisms was not statistically compared
**Ho et al., [42]**<br><br>China<br><br>(follow-up period: 1 year)	Caries-Free/remained caries-free: (18)<br><br>Caries-free/transitioning to caries affected (18)<br><br>Caries affected (18)<br><br>[from 36 months old at baseline to 48 months old at the follow-up]	NR<br>[it is not clear whether non-cavitated lesions were included]	Supragingival plaque (collected after 2 hours of fasting; caries-free: pool of molars and incisors surfaces; caries-affected: plaque collected from enamel surface of carious teeth or from adjacent enamel)	16S- rRNA PacBio [NR]	**Caries-free/remained caries-free:** greater abundance of *Corynebacterium durum, Haemophilus haemolyticus,* human oral bacterium C730, *Prevotella_conceptionensis* at baseline<br>**Caries-free/ transitioning to caries affected:** greater abundance of *Bacteroidetes_bacterium, Bergeyella_sp*, *Campylobacter_showae, Capnocytophaga genosp, Capnocytophaga granulosa, Capnocytophaga_sp_oral_taxon_863_str_F0517, Capnocytophaga_genosp_AHN8471, F. nucleatum, Lachnoanaerobaculum_cf_saburreum_oral_strain_C27KA, L. wadei, L. buccalis, L. genomosp, Leptotrichia_sp_oral_taxon_215, Porphyromonas_sp_oral_taxon_278, Prevotella denticola, Prevotella pallens, Selenomonas flueggei, Selenomonas_sp_oral_taxon_137,*

					Streptococcus_genomosp_C4, S. cristatus, Streptococcus_anginosus, Streptococcus pneumoniae Tannerella_sp_oral_taxon_BU63 at baseline<br>**Caries-affected**: greater abundance of *Capnocytophaga endodontalis, Leptotrichia wadei, Streptococcus mutans* at baseline
**Simon-Soro et al.** [43]<br><br>Scotland<br><br>(follow-up period: 1 year)	Caries-free/remained caries-free (14) [mean of 4.8 ± 0.29 years at baseline]<br><br>Caries-free/transitioning to caries-affected (5) [mean of 4.8 ± 0.44 years at baseline]<br><br>Caries-affected at baseline (14) [mean of 4.5 ± 0.35 years old at baseline]	Caries-free – (dmft=0)<br><br>Caries-free/ transition for caries-affected – Baseline (0), follow-up (dmft = 1.8)<br><br>Caries-affected – Baseline (2.9), follow-up (dmft = 3.9)<br><br>(frank cavitated lesion)	Unstimulated saliva (collected with oral swab)	16rRNA (Universal bacterial primer) 454 pyrosequencing<br><br>qPCR for *S. mutans* quantification<br><br>[>1%]	**Caries-free/remained caries-free**: greater levels of *Rothia* spp. at baseline<br><br>**Caries-free/transitioning to caries-affected**: greater levels of *Streptococcus* spp. at baseline compared with those who remained caries-free.<br><br>At baseline and at the follow-up, for all the three groups, the bacterial composition was dominated by *Streptococcus* species but with a significant decrease in their proportion at follow-up compared with baseline. The levels of *Streptococcus* spp. at follow-up were not different among those who remained caries-free and those who transitioned to caries-affected condition.

**Grier et al.** [44]<br><br>USA<br><br>(follow-up periods: 6, 12, 18 and 24 months)	Caries-Free/remained caries-free (20)<br><br>Caries-free/transitioning to caries affected (ECC: 36) [from1 to 3 years-old]	NR<br><br>(non-cavitated lesion)	Stimulated saliva (collected with a vacuum pump)	16S- rRNA (V1-V3) Illumina MiSeq<br><br>[0.1%]	**Caries-free/remained caries-free:** greater abundance of *Neisseria subflava, Pseudomonas* spp., and *Veillonella parvula* at baseline; Greater abundance of *Oribacterium* spp., *Peptostreptococcus* sp., *Pseudomonas* spp. and *Veillonella parvula* across all follow-up periods.<br><br>**Caries-affected**: greater abundance of *Corynebacterium* spp., *Eikenella* spp., *Kingella* spp., *Neisseria cinerea*, *Rothia mucilaginosa, Prevotella tanaerae, Selenomonas* spp., *Selenomonas noxia,* at baseline. Greater abundance of *Corynebacterium* spp., *Kingella* spp., *Rothia mucilaginosa* and *Selenomonas* spp. across all follow-up periods.
**Dashper et al.** [45]<br><br>Australia<br><br>(follow-up periods: 1.9 ± 0.8, 7.7 ± 1.3, 13.2 ± 1.2, 19.7 ± 2.0,	Caries-free/remained caries-free (89)<br><br>Caries-free/transitioning to caries affected: 12 children at 39 months; 33 children from 39 to 48.6 months old= total of 45 children)	All children were caries-free up to 19.7 months<br><br>**Caries-affected:** mean ECC severity score* of 20.5 ± 11.4 at 39 months old and 17.3 ± 9.0 at 48.6 months old<br><br>* ECC severity score was calculated by multiplying the number of teeth affected in a child by their ICDAS II score and then summing them	Unstimulated saliva (drooling)	16 rRNA (v4) Ion torrent<br><br>[NR]	**Caries-free/remained caries-free:**<br>*At 39 months*: Bergeyella* 602D02, *F. periodonticum*, *L. frumenti, L. salivarius P. propionicus, S. wiggsiae, S. longum*<br>*At 48.6 months: *Capnocytophaga AM420030, Chloroflexi genomosp. P1, Leptotrichia Arg j44, Peptococcus oral taxon 167, Peptostreptococcus stomatis, Porphyromonas CW034, Prevotella oral taxon 299, P. pallens, P. shahii, S. longum.*<br>**Caries-affected:**

39.0 ± 3.2 and 48.6 ± 1.6 months)	[from 1.9 to 48.6 months old]	(cavitated lesions – enamel and dentin)	*At 39 months*: Atopobium parvulum, Lachnospiraceae 502G12, L. shahii, P. melaninogenica, Prevotella oris, P. salivae, Staphylococcus hominis, V. atypica dispar parvula, S. longum, S. mutans, S. vestibularis salivarius.*<br>*At 48.6 months: *Bifidobacterium longum, Fusobacterium nb2727a02c1, Leptotrichia IK040, L. shahii, P. oris, S. wiggsiae, Selenomonas oral taxon 149, S. sputigena, S. mutans, S. longum.*<br><br>There was little difference in the oral microbiomes at 1.9, 7.7 and 19.7 months-of-age of those children who developed disease at the later time points compared with those that remained healthy (data not shown).

*cut-off threshold for the inclusion of taxa on relative abundance data ; Caries affected= non cavitated and/or cavitated lesion; NR= not reported; w.o=without; ECC: early childhood caries; dmfs: number of decayed, missed and filled surfaces; dmft=number of decayed, missed and filled teeth; dt=number of decayed teeth; ds=number of decayed surfaces; dfs=number of decayed and filled surfaces; ICDAS=International Caries Detection and Assessment System

Table S2. Methodological quality assessment of the primary studies based on Newcastle-Ottawa Scale [31]

Studies	Domains								Score
	Selection				Comparability	Outcome			
	1	2	3	4	1	1	2	3	
Kahharova et al., [33]	-	★	★	★	-	★	★	-	FAIR
Dzidic et al., [34]	-	★	★	★	★	★	★	★	GOOD
Zhu et al., [35]	-	★	★	-	★	★	★	★	GOOD
L.Xu et al., [36]	-	★	★	★	★	★	★	★	GOOD
Teng et al., [37]	-	★	★	-	★	★	★	★	GOOD
H.Xu et al., [38]	-	★	★	★	★	★	★	★	GOOD
Kim et al., [39]	-	★	★	★	★	★	★	★	GOOD
Raksakmanut et al., [40]	-	★	★	★	★	★	★	★	GOOD
Yu et al., [41]	-	★	★	-	★	★	★	★	GOOD
Ho et al., [42]	-	★	★	-	★	★	★	★	GOOD
Simon-Soro et al., [43]	-	★	★	-	★	★	★	★	GOOD
Grier et al., [44]	-	★	★	★	★	★	★	★	GOOD
Dashper et al., [45]	-	★	★	★	★	★	★	★	GOOD

Selection: 1) Representativeness of the exposed cohort; 2) Selection of the non-exposed cohort; 3) Ascertainment of exposure; 4) Demonstration that outcome of interest was not present at start of study; Comparability: 1) Comparability of cohorts on the basis of the design or analysis; Outcome: 1) Assessment of outcome; 2) Was follow-up long enough for outcomes to occur; 3) Adequacy of follow up of cohorts;
